# Gingival overgrowth as secondary effect of calcium channel blockers administration. A case report

**Published:** 2014-06-25

**Authors:** M Mironiuc-Cureu, AS Dumitriu, IM Gheorghiu, IM Stoian

**Affiliations:** * Department of Periodontology, Faculty of Dental Medicine, „Carol Davila" University of Medicine and Pharmacy, Bucharest; ** Department of Operative Dentistry, Faculty of Dental Medicine, „Carol Davila" University of Medicine and Pharmacy, Bucharest; *** Department of Biochemistry, Faculty of Medicine, „Carol Davila" University of Medicine and Pharmacy, Bucharest

**Keywords:** Gingival overgrowth, calcium channel blockers, gingivectomy

## Abstract

Abstract

Gingival overgrowth is, among other things, a side effect of the administration of dihydropyridine antihypertensives, generally associated with irritant factors of marginal periodontium. This case refers to a patient, female, who developed a large gingival enlargement that has a combined etiology: the systemic medication with lercanidipina and the presence of dental bridges, which are incorrectly adjusted to the dental cervix. The treatment for this case, involved a complex local treatment (antimicrobial, surgical, endodontic and prosthetic) and the collaboration with a specialist cardiologist. Maintaining the normal gingival parameters in time depends on the possibility of changing the antihypertensive medication, the accuracy of the new dental bridges and the periodic monitoring of the patient.

## Introduction

Gingival overgrowth (gingival hyperplasia) has a multiple etiology, starting with a bacterial origin which is associated with various local factors (decays, unadjusted prosthesis, malocclusions), and continuing with those which appear in the systemic diseases (diabetes, leukemia, immunodeficiency disease) or as a side effect of certain medications (calcium channel blockers, phenytoin, cyclosporine). The idiopathic hyperplasia (gingival elephantiasis). Quite common is the gingival hyperplasia as a side effect of drugs administration of calcium antagonists, because the incidence of hypertension is higher in patients aged three, and, in their treatment, drugs such as calcium channel blockers are often used.

 Many authors have cited the involvement of calcium antagonists in gingival hyperplasia pathogenesis by changes in both the epithelium and the chorion. The epithelial hyperplasia is not caused by the proliferation of keratinocytes but by increasing their lifetime with decreasing apoptosis. Connective tissue accumulates collagen components by decreasing phagocytic activity reducing collagen degradation, which are closely correlated with the accumulation of type I collagen [**1**].

 The size of hyperplasia depends on many factors such as time of drug administration, the daily dose, the combination with other factors, local irritation and individual host response. Calcium antagonists with mainly vascular effects cause most common gingival hypertrophy, and one of these is nifedipine which produces gingival hyperplasia at a dose of 10 mg daily, because its accumulation in gingival sulcus is of 15-316 times more than in the blood.

## Material, Methods

**Fig. 1:**
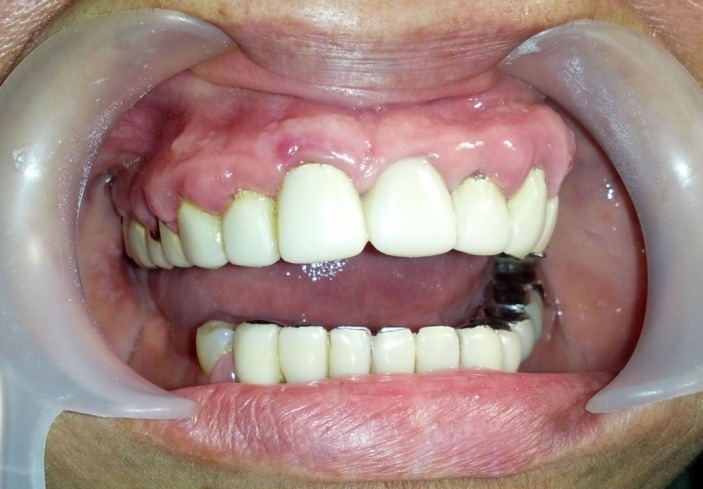
Initial aspects of gingiva

The patient presented in this article is 55 years old, female, diagnosed with hypertension, with a history of stroke (2009). Its antihypertensive medication included Nebilet, Leridip, Noliterax alongside, Sortis and Aspenter. Among these Leridip products (lercandipinum) there is a calcium channel blocker from the group of dihydropyridines, a daily dose of 10 mg being administered for 4 years. In combination with the presence of fixed prosthetic teeth, incorrectly adjusted at the dental cervix, this drug leads to the development of an extensive gingival overgrowth interesting interdental papilla, free gingival margin and partially fixed gingiva (**Fig. 1**). 

**Fig. 2:**
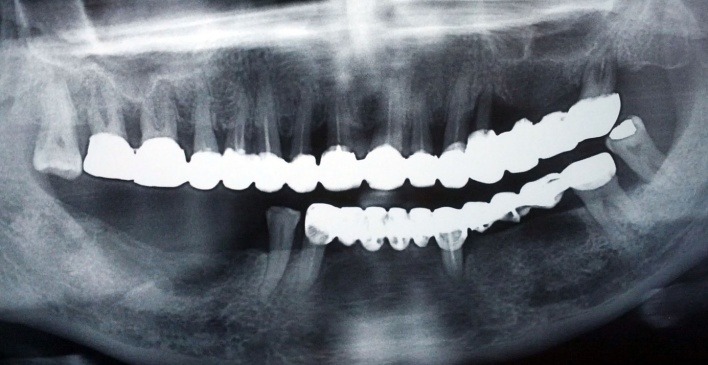
Radiographic aspects

 At presentation, periodontal indices had the following values: PI = 61.61%, TI = 45%, BI = 36.34%. parodontometry showed deep pockets between 3 to 7.5 mm. The panoramic radiography showed lack of adaptation to the dental crowns, and a vertical bone resorption especially in the upper front teeth and 2.4. (**Fig. 2**).

**Fig. 3:**
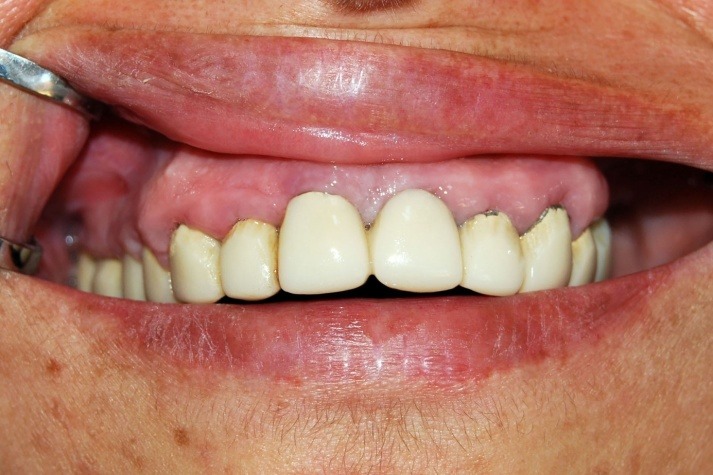
Gingival aspects after initial therapy

 Initial local therapy consisting in supra and subgingival scaling, repeated local applications with antibiotics paste (tetracycline and metronidazole) were associated with general treatment with antibiotics (Amoxicillin + Metronidazole 250mgx3/day 500mgx3/day for 7 days). We obtained a reduction of inflammation with gingival color normalization, reducing gingival bleeding and volume (**Fig. 3**). 

It was decided to proceed to gingivectomy on the upper jaw to remove the gum in excess, in two sessions, one for each half of the upper jaw.

**Fig. 4:**
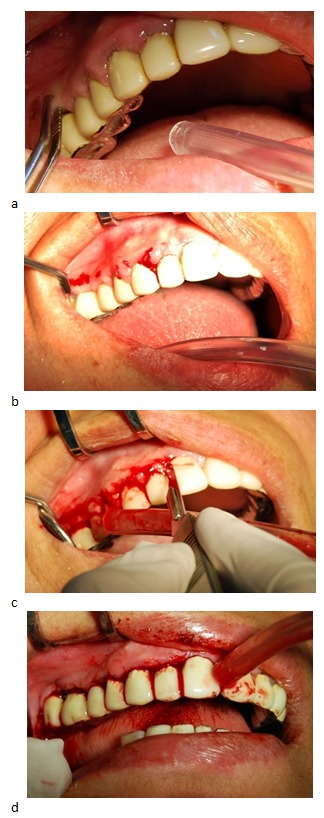
a,b,c,d Surgical aspects on the right side

 We started with quadrant I. After a preliminary pocket depth marking with forceps Krane Kaplan, we performed a horizontal external bevel incision, followed by secondary incisions in sulcus. We removed gum hyperplasia, proceed to gingival curettage, and root planning. After lavage with saline, we protected the wound by periodontal dressing. This was removed after 48 hours (**Fig. 4 a,b,c,d**). 

**Fig. 5:**
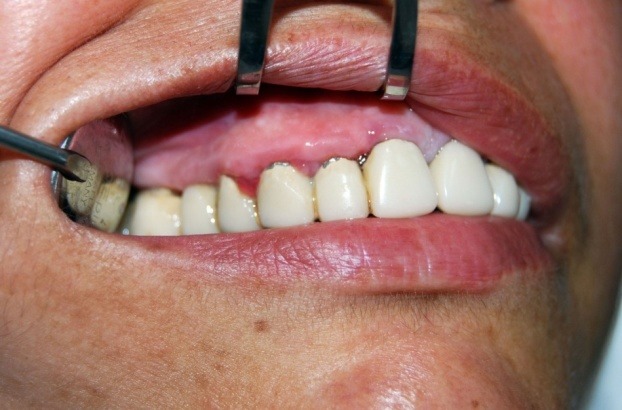
Gingival aspects after 4 weeks from surgery

 In Fig. 5 we can observe gingival aspect after one month from surgery, keeping the normal appearance and color of the gums, with a slight granulation tissue between 1.3 and 1.4 due to retentive dental bridge at that level.

**Fig. 6:**
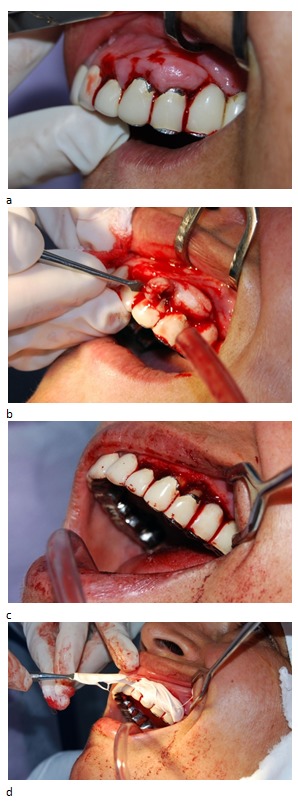
a,b,c,d Surgical aspects on the left side

 After 4 weeks, the same intervention was done in quadrant II (**Fig. 6 a,b,c,d**). 

## Results

The lack of periodontal pockets, resulting in a better gingival aspect, with normal color, volume and consistency was obtained.

After 3 weeks from the second gingivectomy, the patient was sent to prosthetist to remove the dental bridge and restore it. Tooth no 2.4 extraction was necessary due to advanced bone resorption and endodontic treatments were restored. The prosthetist decided to make an acrylic temporary bridge with supragingival margins for a period of 3 months to stabilize the gingiva and restore the alveolar ridge after extraction. After this, the prosthetist will make the final dental bridge.

 To avoid the relapse we recommended a visit at the cardiologist, to change the antihypertensive medication, by replacing the calcium channel blockers with other antihypertensive drugs of a different class.

## Discussion

Calcium antagonists are substances that inhibit the flow of calcium ions through the slow channel membrane. The therapeutic consequences are the inhibition of myocardial contraction, depression of myocardial function, specifically generating potential slow (sinus and atrioventricular node, antiarrhythmic effect, bradycardia) and relaxation of smooth muscle, particularly in the vessels, with vasodilator effect [**2**].

 Calcium antagonists are classified into two categories, each having in principal vascular effects (Nifedipine, Nimodipine, Nicardipine, Flodipina, amlodipine), and the other having main effects on heart (Verapamil, Diltiazem).

 From the pharmacological point of view, they are classified as family dihydropyridine (prototype - Nifedipine), non-dihydropyridine family- in fact phenylalkylamine (Verapamil) and family benzothiazepine (Diltiazem).

 Among these groups of calcium channels blockers, the dihydropyridine family is responsible for gingival enlargement. Most studies have been done on nifedipine, which seems to cause gingival hyperplasia most frequently. Increase of gingival tissues can be observed after 20 days from the administration of nifedipine and gingival mass is constant after 70 days. Gingival enlargement has also been measured in treatments with amlodipine and lercanidipine. It seems that isradipine causes no gingival hyperplasia.

 The volume of hyperplasia depends on the daily dose (more than 10mg), the time of administration, gender (men are more likely than women) and the presence of plaque and local irritation factors (decays, the incorrectly dental bridges) [**3**].

 The clinical aspects include enlargement of the papillae, which take the appearance of masses of connective tissue, which can reach the occlusal plane. Gum may have a nodular appearance, color ranging from red-violet to red-congestion. Rarely, epithelial junction can migrate to the apical, and give birth to real periodontal pockets.

 On histological cups, we found the dystrophia in epithelium, cell segregation and acanthosis in the stratum spinosum, hyperkeratosis and parakeratosis, the presence of fibrous bands in chorion, dilated blood vessels, with teleangiectazic aspect, rich limfoplasmocitar inflammatory infiltrate in gingival chorion [**4**].

 It has been shown that nifedipine can induce changes on Keratinocyte growth factor (KGF) and its receptor (KGFR). Epithelial keratinocytes and mesenchymal fibroblasts may interplay to dynamically regulate gene expression, which may have an effect on the gingival condition following treatment with nifedipine [**5**].

 It has also been suggested that collagen fibers accumulation can be attributed to a decrease of destroying rather than an increase in synthesis [**6**]. Enzymes such as collagenase and proteolytics from the metalloproteinase family predominantly control lifetime and mechanism of collagen structural remodeling. Members of this family of enzymes may specifically trigger digestion of collagen but also of the structural components of the extracellular matrix. Maturation of collagen fibers was observed while coloring varies due to the development in green, yellow, orange and red. The existence of type III collagen was observed, it is considered an immature form of collagen that participates in tissue remodeling.

 The case report we presented in this article showed gingival hyperplasia when administered 10 mg daily Leridip (Lercandipinum) along with Nebilet and Noliterax in a patient. In addition, prosthetic restorations, especially the upper arch were not correctly adapted to the tooth cervix. Also the aesthetically compounds (acrylate) present lack of finishing and polishing at the margins. From the X-ray it can be observed that bone loss is quite advanced, indicating a deep chronic marginal periodontitis associated with gingival overgrowth due to lercandipine (second generation of dihydropyridine).

## Conclusions

 Given that the prevalence of gingival hyperplasia as a side effect of antihypertensive medication with calcium antagonists is 30-50%, it requires an assessment of periodontal status before prescribing them, and after the substance accumulates in the body. It is better to use other antihypertensive calcium antagonists in case of a pre-existing periodontium disease. However, if their use is necessary, patients should be called for a dental visit at every 6 months for a proper debridement. 

 It is equally important to evaluate any existing prosthetic restorations, the dental fillings and other factors that may influence the volume of the local gingival hyperplasia.

 In hypertensive patients with risk of gingival overgrowth, it is advisable to have a collaboration between the dentist and the cardiologist to maintain the periodontal health and improve the quality of the patients’ life.
